# Healthcare costs and utilization for privately insured patients treated for non-infectious uveitis in the USA

**DOI:** 10.1186/1869-5760-3-64

**Published:** 2013-11-06

**Authors:** David S Chu, Scott J Johnson, Usha G Mallya, Matthew R Davis, Rachael A Sorg, Mei Sheng Duh

**Affiliations:** 1 Institute of Ophthalmology and Visual Science, New Jersey Medical School, Rutgers University, 90 Bergen Street, Suite 6100, Newark, NJ 07960, USA; 2 Metropolitan Eye Research and Surgery Institute, Palisades Park, NJ, USA; 3 Analysis Group, Inc, 111 Huntington Avenue #10, Boston, MA 02199, USA; 4 Novartis Pharmaceuticals Corporation, 1 Health Plaza, East Hanover, NJ 07936, USA

**Keywords:** Comorbidities, Healthcare costs, Healthcare utilization, Uveitis

## Abstract

**Background:**

The purpose of this study was to describe comorbidities, healthcare costs, and resource utilization among patients with chronic non-infectious uveitis initiating corticosteroid, immunosuppressants, or biologics.

In this retrospective cohort study, patients with a non-infectious uveitis diagnosis and continuous insurance coverage during a 6-month baseline were selected from a privately insured claims database with 80.7 million enrollees. Index dates were defined as the first prescription/administration of a corticosteroid, immunosuppressant, or biologic between 2003 and 2009. Comorbidities, healthcare costs, and utilization were analyzed in a per-member-per-month (PMPM) framework to account for varying between-patient treatment periods, defined as continuous medication use within the same class. Wilcoxon rank-sum and chi-square tests were used for comparisons of costs and categorical outcomes.

**Results:**

Patients on corticosteroids (*N* = 4,568), immunosuppressants (*N* = 5,466), and biologics (*N* = 1,694) formed the study population. Baseline PMPM inpatient admission rates were 0.029 for patients on corticosteroids, 0.044 for patients on immunosuppressants, and 0.045 for patients on biologics (*p* < 0.001 immunosuppressants or biologics versus corticosteroids); during treatment, PMPM inpatient admissions increased to 0.044 and 0.048 for patients taking corticosteroids and immunosuppressants, respectively, but decreased to 0.024 for patients taking biologics (*p* < 0.001 versus corticosteroids and *p* = 0.003 versus immunosuppressants). Baseline average PMPM costs for patients taking corticosteroids, immunosuppressants, and biologics were US$935, US$1,738, and US$1,439 (*p* < 0.001 between groups), while on-treatment PMPM costs excluding drug costs increased to US$1,129 for patients taking corticosteroids but lowered to US$1,592 for patients taking immunosuppressants, and US$918 for patients taking biologics (*p* < 0.001 versus corticosteroids or immunosuppressants).

**Conclusions:**

There is significant economic burden associated with existing treatments of uveitis. Corticosteroids may be overused as a treatment for uveitis.

## Background

Uveitis is an inflammatory condition of the eye that is typically characterized by redness, pain, light sensitivity, and blurred/decreased vision and is associated with numerous ocular diseases and systemic conditions. This inflammation can also be categorized by the location of inflammation, including anterior uveitis (e.g., iritis), intermediate uveitis (e.g., pars planitis, vitritis), and posterior uveitis (e.g., choroiditis, retinitis). Anterior uveitis is the most common form of uveitis in most populations, particularly in Western countries, accounting for about 50% to 60% of all uveitis cases in most tertiary referral centers and around 90% in primary care settings [1]. The majority of uveitis-related visual morbidity occurs in patients with posterior segment uveitis, which includes intermediate, posterior, and panuveitis.

Uveitis is responsible for an estimated 10% of cases of blindness in the USA [2,3], including 30,000 new cases of legal blindness each year [4]. Uveitis is a major cause of visual morbidity in the working age group [5]. Early diagnosis and treatment are important to prevent the vision-threatening complications of uveitis, including cataract, glaucoma, retinopathy, and macular edema [6]. Goals of treatment include suppressing inflammation and achieving remission [7].

Three drug classes that constitute the primary treatment modalities for uveitis include corticosteroids, traditional (non-biologic) immunosuppressive agents, and biologics. Corticosteroids are typically used as the first-line drug therapy for non-infectious inflammatory conditions [8]. They may be used topically, administered systemically via oral, intravenous or intramuscular route, or injected periocularly or implanted surgically. Serious side effects such as hypertension, cardiac failure, weight gain, osteoporosis, myopathy, osteonecrosis, and gastrointestinal side effects [9] are associated with the chronic use of systemic corticosteroids. If a patient's uveitis is not completely quiet after several weeks of high-dose corticosteroids and maintained with 10% mg per day of prednisone (or equivalent) within 3% months or if the posterior segment is being affected, established guidelines recommend the use of steroid-sparing agents [10], such as second-line traditional immunosuppressant therapy (i.e., antimetabolites, T cell inhibitors and alkylating agents) [8,11].

For patients whose uveitis condition is refractory to traditional immunosuppressants, biologic therapies may be considered. Examples of biologics used as a third-line therapy include tumor necrosis factor (TNF) inhibitor agents, such as infliximab and adalimumab; interferons, such as recombinant human IFN-α-2a and IFN-α-2b; and anti-interleukin therapy [12]. Even though there have been a large number of reports on the use of biologic therapies to treat uveitis, there have been no controlled trials comparing the efficacy of different biologic therapies with each other or with traditional immunosuppressants, and further research is needed to support clinical decisions regarding choice of agent, time of initiation, and course of therapy [12].

Claims data analyses provide large samples for empirical analyses, which could contribute to understanding the economic burden related to uveitis as well as indicators for ophthalmologic comorbidities. Previous research has examined the incidence and prevalence of uveitis in a privately insured population in Northern California and Medicare populations [13,14]. Gritz and Wong [13] found that the incidence of uveitis was higher than results from smaller previous studies, the prevalence increased with age, and women had a higher prevalence of uveitis than men. Reeves et al. [14] found that the burden of uveitis is higher in an elderly population than previous research had indicated.

In comparison to previous studies, the current study utilizes pharmacy and non-elderly claims excluded in the analysis by Reeves et al. [14] and includes a larger sample to describe disease burden related to and treatment patterns for patients receiving treatment for uveitis. Specifically, we examine baseline characteristics, uveitis-related ophthalmologic outcomes, healthcare utilization, and healthcare costs among patients receiving the three treatment options (i.e., corticosteroids, immunosuppressive agents, and biologics) with non-infectious uveitis in a privately insured population. We use a large, longitudinal claims data as an efficient way to study uncommon diseases like non-infectious uveitis [15].

## Methods

### Data and study design

The Thomson Reuters MarketScan® Commercial Databases, which have been used previously to estimate the economic burden of ophthalmologic disease [16], was used in this analysis. This database contains individual-level claims and enrollment data for approximately 80.7 million members from January 2003 to October 2009. The data include the enrollment history, demographics, medical claims, and pharmacy claims for employees, dependents, and retirees in the USA with primary insurance coverage.

We used a retrospective cohort design to address our research questions. Retrospective cohorts using claims data can provide equally valid results as a prospective cohort study while being more economical [17].

### Patient selection

Prior literature was used to identify codes for non-infectious uveitis using the International Classification of Diseases, Ninth Revision, Clinical Modification (ICD-9-CM) coding system. Diagnosis codes including both posterior and anterior disease were used in the base case (Additional file 1: Table S1 for a complete list of ICD-9-CM codes). In a sensitivity analysis, codes more specific to posterior disease were used.

Patients were included in the sample if they had at least one claim for non-infectious uveitis diagnosed by an ophthalmologist (or optometrist) or two claims diagnosed by other physician specialists. Patients were also required to have at least one prescription dispensing or administration of corticosteroids, immunosuppressants, or biologics on or after their diagnosis of non-infectious uveitis (the list of drugs included can be found in Additional file 1: Table S2). In order to exclude patients with post-surgical inflammation, patients with incisional intraocular surgery within 3% months of their uveitis diagnosis were excluded.

**Figure 1:**
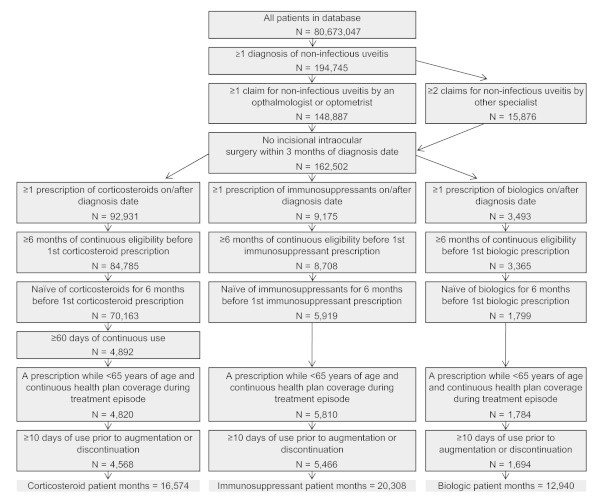
Sample selection criteria and sample development.

Patients were also required to have at least 6% months of continuous insurance coverage prior to their first prescription or administration, which was considered their baseline period, as well as at least 10% days of study period drug use. In order to identify patients with chronic uveitis, patients receiving corticosteroids had to have continuous prescriptions for 60 or more days. Sensitivity analyses requiring 90 or 30 or more days were implemented. Patients over 65% years of age were excluded from the analysis to avoid incomplete claims due to Medicare dual coverage. Figure 1 contains additional detail on patient selection.

Three samples were developed, including patients receiving their first dose of corticosteroid therapy only (CTS), traditional immunosuppressants/immunomodulators (IMS) with or without corticosteroids, and biologics (BIO) with or without corticosteroids or immunosuppressants. Patients' first date of therapy was considered their index date. Additionally, patients were required to have no evidence of their sample's respective drug therapy in the 6% months prior to their index date.

Patient gender, age, health insurance type, and index year were reported. To ascertain general comorbidity burden of the patients at baseline, we used the Deyo adaptation of the Charlson comorbidity index, excluding ophthalmologic diagnosis codes [18].

### Treatment periods

Analyses were performed based on treatment periods, which form the study period in the analysis. Treatment periods were defined as the continuous on-therapy period for each patient within the same class, spanning from a patient's index date to the date of either stopping therapy, stepping up therapy, or being censored in the data (e.g., disenrollment from health plan). Stopping therapy was based on the patient not refilling the prescription for a period of days equal to 150% the number of days in the previous prescription, based on the days supply variable in the pharmacy claims for prescription drugs [19] and based on the literature for physician-administered intramuscular injectables and infusions [12]. For example, if a patient received a first prescription of prednisone on 01 April 2007 with a 30% days' supply, then a second 30-day script on May 16 (which is a treatment gap of 15% days or 50% of the days’ supply of the preceding script), and then no further prescriptions, a steroid episode of 30 + 15 + 30% days would be assumed.

Stepping up therapy ended a treatment episode and occurred on the day that a patient received a prescription or administration for a higher class of therapy (e.g., from corticosteroids to immunosuppressants or immunosuppressants to biologics). Switching within the same class (e.g., from one immunosuppressant agent to another) did not end a treatment episode.

### Outcomes

Healthcare costs and utilization were measured in a per-member-per-month (PMPM) framework to standardize estimates across patients with treatment periods of different lengths. Fractional months were grossed up to complete months based on a patient's observed treatment days.

Healthcare costs were measured using the paid amount by insurers for patient's covered benefits. Categories of costs include costs related to inpatient care, emergency room (ER) care, office or outpatient (OP) care, and care for each type of therapy. Costs were inflation adjusted to 2009 dollars based on the medical consumer pricing index. Utilization was measured in terms of monthly inpatient stays per patient and inpatient days per hospitalized patient, number of ER visits, number of OP visits, and number of prescription drug scripts.

Kaplan-Meier analysis was used to assess time to augment or switch to a next-step class of therapy. Augmenting therapy was considered to be adding a new class of therapy before the current treatment episode had ended. Switching therapy was based on beginning a new class of therapy at any time observable in the data before a censoring event, including loss of eligibility or the last available data in September 2009.

Though not a primary focus of the analysis, ophthalmologic outcomes in terms of counts of diagnoses codes for retinal detachments (ICD-9-CM codes: 361.0-361.3, 361.8, 361.9), glaucoma (ICD-9-CM codes: 365.0-365.6, 365.8, 365.9), cataracts (ICD-9-CM codes: 366.0-366.5, 366.8, 366.9), cystoid macular degeneration (ICD-9-CM 362.53), visual disturbances (ICD-9-CM codes: 368.1, 368.3-368.6, 368.8, 368.9), blindness (ICD-9-CM codes: 369.0-369.4, 369.6-369.9), and other visual complications including phthisis bulbi, chorioretinal scars, other macular scars, optic atrophy, optic neuritis, other disorders of optic nerve, hypotony, and band-shaped keratopathy (ICD-9-CM codes: 360.4, 363.3, 363.32, 377.1, 377.3, 377.4, 360.3, and 371.4) were also reported. Additional file 1: Table S3 contains additional detail.

### Sensitivity analysis

Comparison of baseline and study period all-cause costs were made for the CTS, IMS, and BIO samples excluding patients with rheumatoid arthritis, Crohn's disease, psoriasis, or ankylosing spondylitis based on their ICD-9-CM diagnosis codes in the baseline or study period. Patients with these conditions may receive any of these treatments, so we excluded them in a sensitivity analysis.

Posterior uveitis may be more severe than anterior disease, though some codes may describe both types of disease. In a second sensitivity on all-cause costs, we include patients using a subset of ICD-9-CM codes that are more specific to posterior disease (see Additional file 1: Table S1). Lastly, we also assessed results excluding diagnoses made only by non-ophthalmologists in order to increase the accuracy of the non-infectious uveitis diagnosis.

### Statistical analysis

All analyses were conducted using SAS version 9.2 (SAS Institute Inc., Cary, NC, USA). Statistical comparisons were conducted using chi-square tests for categorical outcomes and Wilcoxon rank-sum tests for continuous outcomes within baseline and study periods. Wilcoxon signed-rank tests assessed differences in pre- versus post-comparisons.

## Results

The distribution of the study population was 38.9% CTS (*N* = 4,568), 46.6% IMS (*N* = 5,466), and 14.4% BIO (*N* = 1,694) out of 11,728 patients. The study periods were 16,574, 20,308, and 12,940 patient-months for the CTS, IMS, and BIO groups. Approximately 93% of the CTS were (65,271 of 70,163) patients excluded from the CTS sample as a result of patients having fewer than 60 continuous days of corticosteroids prescriptions.

As shown in Table 1, about 84% (9,808 of 11,728 total patients) of patients were between age 35 and 64 at their index date. A higher proportion of women receive IMS (72.1%, 3,940 of 5,466 IMS patients) than BIO (61.4%, 1,040 of 1,694 BIO patients) or CTS (59.7%, 2,729 of 4,568 CTS patients) (*p* < 0.0001 for IMS versus CTS or BIO). CTS patients were more likely to be enrolled in an HMO (*p* = 0.0001 for CTS vs. IMS; *p* = 0.0004 for CTS versus BIO). The numbers of patients diagnosed has grown over time, with 2009 reflecting only 9% months of available data. Between therapies, there were no apparent trends related to the year in which therapy was prescribed. In other words, neither IMS nor BIO therapy shows an increase in the rate of use for patients fitting the selection criteria over the period from 2003 to 2009.

**Table 1:** Baseline demographics for non-infectious uveitis patients by treatment groups

	**CTS patients**^**a**^ **(*N*** **= 4,568)**	**IMS patients**^**b**^ **(*N*** **= 5,466)**	**BIO patients** **(*N*** **= 1,694)**
Gender, *N* (%)			
Male	1,839 (40.3%)	1,526 (27.9%)^a^	654 (38.6%)^b^
Female	2,729 (59.7%)	3,940 (72.1%)^a^	1,040 (61.4%)^b^
Age, *N* (%)			
0–17,%	162 (3.5%)	350 (6.4%)^a^	153 (9.0%)^a,b^
18-24	142 (3.1%)	170 (3.1%)^a^	77 (4.5%)^a,b^
25-34	305 (6.7%)	388 (7.1%)^a^	173 (10.2%)^a,b^
35-44	704 (15.4%)	868 (15.9%)^a^	390 (23.0%)^a,b^
45-54	1,360 (29.8%)	1,683 (30.8%)^a^	472 (27.9%)^a,b^
55-64	1,895 (41.5%)	2,007 (36.7%)^a^	429 (25.3%)^a,b^
Index year, *N* (%)			
2003	175 (3.8%)	219 (4.0%)	72 (4.3%)
2004	401 (8.8%)	507 (9.3%)	151 (8.9%)
2005	641 (14.0%)	723 (13.2%)	208 (12.3%)
2006	730 (16.0%)	803 (14.7%)	250 (14.8%)
2007	862 (18.9%)	1,088 (19.9%)	346 (20.4%)
2008	1,063 (23.3%)	1,280 (23.4%)	420 (24.8%)
2009	696 (15.2%)	846 (15.5%)	247 (14.6%)
Health insurance type, *N* (%)			
HMO	924 (20.2%)	924 (16.9%)^a^	282 (16.6%)^a^
PPO	2,456 (53.8%)	3,216 (58.8%)^a^	1,014 (59.9%)^a^
EPO	18 (0.4%)	34 (0.6%)^a^	7 (0.4%)^a^
Indemnity	72 (1.6%)	94 (1.7%)^a^	29 (1.7%)^a^
Other	1,098 (24.0%)	1,198 (21.9%)^a^	362 (21.4%)^a^

HMO, health maintenance organization; PPO, preferred provider organization; EPO, extended provider organization. Chi-square tests were used to test differences in categorical variables. ^a,b^Data with superscripted letter(s) indicate a significant difference (*P* < 0.05) from the uveitis subtype represented by that letter.

As shown in Table 2, in the 6% months prior to initiating therapy, BIO and IMS patients were associated with the highest Charlson comorbidity index score. IMS patients were associated with the highest level of glaucoma, cataracts, and visual disturbances. Both IMS and BIO patients had greater baseline rates of cystoid macular degeneration than did CTS patients.

**Table 2:** Baseline ophthalmologic comorbidities, utilization, costs, and prior therapy for patients with non-infectious uveitis by treatment group

	**CTS patients**^**a**^ **(*N*** **= 4,568)**	**IMS patients**^**b**^ **(*N*** **= 5,466)**	**BIO patients** **(*N*** **= 1,694)**
Patient months	27,408	32,796	10,164
Baseline monthly ophthalmologic comorbidities, mean (std)			
Glaucoma	0.041 (0.15)	0.045 (0.16)	0.028 (0.13)^a,b^
Cataract	0.022 (0.09)	0.024 (0.10)	0.016 (0.08)^a,b^
Cystoid macular degeneration	0.004 (0.03)	0.008 (0.06)^a^	0.008 (0.07)^a^
Retinal detachments	0.006 (0.05)	0.007 (0.06)	0.005 (0.06)^a,b^
Visual disturbances	0.005 (0.04)	0.007 (0.05)^a^	0.006 (0.05)
Blindness	0.001 (0.02)	0.002 (0.02)^a^	0.002 (0.02)^a^
Other visual complications	0.012 (0.06)	0.024 (0.11)^a^	0.019 (0.11) ^b^
Baseline Charlson Comorbidity Index (CCI), mean (std)	0.482 (1.06)	0.730 (1.29)^a^	0.802 (1.15)^a,b^
Baseline monthly comorbidities, mean (std)			
Crohn's disease	0.009 (0.10)	0.024 (0.22)^a^	0.086 (0.43)^a,b^
Rheumatoid arthritis	0.010 (0.09)	0.036 (0.18)^a^	0.125 (0.33)^a,b^
Psoriasis	0.005 (0.10)	0.017 (0.18)^a^	0.054 (0.31)^a,b^
Ankylosing spondylitis	0.005 (0.06)	0.013 (0.10)^a^	0.095 (0.23)^a,b^
Baseline monthly total healthcare costs, mean [median] (std) (US$)	935 [279] (2,840)	1,738 [492] (5,763)^a^	1,439 [597] (3,171)^a,b^
Medical services	735 [134] (2,741)	1,497 [288] (5,668)^a^	1,204 [375] (3,039)^a,b^
Inpatient stay (IP)	294 [0] (2,177)	692 [0] (4,816)^a^	408 [0] (2,233)^a^
Emergency room (ER)	18 [0] (119)	24 [0] (142)^a^	26 [0] (113)^a,b^
Office/outpatient (OP)	423 [123] (1,041)	782 [263] (1,787)^a^	771 [337] (1,528)^a,b^
Pharmacy services	200 [66] (453)	241 [87] (483)^a^	234 [104] (485)^a,b^
Corticosteroids	0 [0] (0)	3 [0] (9)^a^	3 [0] (11)^a^
Immunosuppressants	9 [0] (82)	0 [0] (0)^a^	20 [0] (104)^a,b^
Biologics	24 [0] (193)	48 [0] (271)^a^	0 [0] (0)^a,b^
Baseline monthly total healthcare resource utilization			
Inpatient stay (IP)			
Number of patients, *N* (%)	64 (1.4%)	114 (2.1%)^a^	39 (2.3%)^a^
Number of stays/patient, mean (SD)	0.029 (0.19)	0.044 (0.22)^a^	0.045 (0.19)^a^
Number of days/ hospitalized patient, mean (SD)	3.88 (8.05)	4.133 (5.76)	3.908 (4.03)^a^
Emergency room (ER)			
Number of patients, *N* (%)	89 (2.0%)	132 (2.4%)^a^	46 (2.7%)^a^
Number of visits/patient, mean (SD)	0.028 (0.10)	0.035 (0.11)^a^	0.042 (0.12)^a^
Office/outpatient (OP)			
Number of patients, *N* (%)	722 (15.8%)	900 (16.5%)^a^	281 (16.6%)^a,b^
Number of visits/patient, mean (std)	1.426 (1.48)	2.172 (1.82 )^a^	2.372 (1.82)^a,b^
Baseline treatment characteristics (over the 6 month period), *N* (%)			
Baseline any corticosteroid use	--	3,784 (69.2%)^a^	1,217 (71.8%)^a,b^
Baseline any immunosuppressant use	280 (6.1%)	--	652 (38.5%)^a,b^
Baseline any biologic use	112 (2.5%)	313 (5.7%)^a^	--

CTS, corticosteroids; IMS, immunosuppressive therapy; BIO, biologics. Chi-square tests were used to test differences in categorical variables. Wilcoxon tests were used to test differences in continuous variables. ^a,b^Data with superscripted letter(s) indicate a significant difference (*P* < 0.05) from the uveitis subtype represented by that letter.

Small proportions of CTS patients had evidence of prior IMS (6.1%, 280 of 4,568 CTS patients) or BIO (2.5%, 112 of 4,568 CTS patients) use before their index CTS dose, indicating that the sample was not completely naïve to therapy. Most IMS patients received steroids (69.2%, 3,784 of 5,466 IMS patients), and some IMS patients had received biologics (5.7%, 313 of 5,466 IMS patients). Most BIO patients had received steroids (71.8%, 1,217 of 1,694 BIO patients), and more than one-third received IMS (38.5%, 652 of 1,694 BIO patients). These treatment patterns are consistent with step therapy, where fewer patients advance to higher levels of therapy.

During the baseline period, IMS patients were associated with the highest monthly all-cause costs (US$1,738 per month) followed by BIO (US$1,439) and CTS patients (US$935) (*p* < 0.0001 between each). Inpatient costs represented approximately 31.5% (US$294 of US$935), 39.8% (US$692 of US$1,738), and 28.4% (US$408 of US$1,439) of total costs in the CTS, IMS, and BIO samples.

Monthly rates of hospitalization and emergency room visits in the 6%months prior to initiating therapy were significantly different among the therapy groups. In terms of patients who had any inpatient stay, BIO patients were associated with the highest proportion of patients hospitalized per month (2.3%, 39 of 1,694 BIO patients), followed by IMS patients (2.1%, 114 of 5,466 IMS patients) and CTS patients (1.4%, 64 of 4,568 CTS patients) (*p* < 0.0001 for IMS and BIO versus CTS). BIO patients were associated with the highest proportion of patients going to the ER per month (2.7%, 46 of 1,694 BIO patients) followed by IMS patients (2.4%, 132 of 5,466 IMS patients) and CTS patients (2.0%, 89 of 4,568 CTS patients).

Though not a primary focus of our analysis, ophthalmologic comorbidities per patient in the study period are presented in Table 3. CTS patients were associated with a higher level of diagnoses of glaucoma, cystoid macular degeneration, and retinal detachments compared to IMS or BIO. CTS patients were also associated with a higher level of visual disturbances compared to IMS. Incidence of claims among CTS patients increased in the study period relative to the baseline in glaucoma, cystoid macular degeneration, retinal detachments, and visual disturbances; however, incidence of claims among IMS and BIO patients decreased or remained about the same in every category except for glaucoma, where it increased for both.

**Table 3:** Study period ophthalmologic comorbidities and prior treatment for patients with non-infectious uveitis by treatment group

	**CTS patients**^**a**^ **(*N*** **= 4,568)**	**IMS patients**^**b**^ **(*N*** **= 5,466)**	**BIO patients** **(*N*** **= 1,694)**
Patient months	16,574	20,308	12,940
Mean months/treatment episode (time to discontinuation), mean (std)	3.63 (3.13)	3.72 (5.61)^a^	7.64 (9.36)^a,b^
Study period ophthalmologic comorbidities, mean (std)			
Glaucoma	0.058 (0.22)	0.052 (0.22)^a^	0.034 (0.14)^a^
Cataract	0.022 (0.12)	0.025 (0.11)	0.016 (0.07)
Cystoid macular degeneration	0.013 (0.10)	0.010 (0.07)^a^	0.005 (0.04)^a^
Retinal detachments	0.011 (0.10)	0.004 (0.05)^a^	0.002 (0.03)^a^
Visual disturbances	0.007 (0.07)	0.004 (0.04)^a^	0.003 (0.02)
Blindness	0.001 (0.03)	0.002 (0.03)	0.000 (0.01)
Other visual complications	0.011 (0.09)	0.015 (0.12)	0.007 (0.05)
Augmenting or switching			
Augmented to immunosuppressants, *N* (%)	348 (7.60%)		
Mean days to augmenting, conditional on augmenting, mean (std)	57 (56)		
Augmented to biologics, *N* (%)	96 (2.10%)	346 (6.30%)	
Mean days to augmenting, conditional on augmenting, mean (std)	42 (37)	78 (116)^a^	
Switched to immunosuppressants, *N* (%)	615 (13.50%)		
Mean days to switching, conditional on switching, mean (std)	213 (299)		
Switched to biologics, *N* (%)	221 (4.80%)	615 (11.30%)	
Mean days to switching, conditional on switching, mean (std)	256 (358)	213 (295)	

CTS, corticosteroids; IMS, immunosuppressive therapy; BIO, biologics. Chi-square tests were used to test differences in categorical variables. Wilcoxon tests were used to test differences in continuous variables. ^a,b^Data with superscripted letter(s) indicates a significant difference (*P* < 0.05) from the uveitis subtype represented by that letter.

In the study period, most CTS patients (72%, 3,288 of 4,568 CTS patients) neither augmented nor switched to IMS or BIO, and most IMS patients (82%, 4,505 of 5,466 IMS patients) neither augmented nor switched to BIO.

Study period utilization and costs appear in Table 4. As shown in Table 2, CTS patients were associated with the lowest number of monthly ER visits and inpatient stays per patient in the 6% months prior to their index date. In the study period, IMS patients were had the highest number of inpatient stays per patient. BIO patients had the highest number of inpatient stays and ER visits at baseline. However, in the post-index period, BIO patients had the lowest number of inpatient stays or ER visits. The BIO group was the only group to have a reduction in both the number of inpatient stays and ER visits. Monthly inpatient stays decreased by 0.021 (*p* = 0.0033) stays per patient and ER visits decreased by 0.012 (*p* = 0.0031) visits per patient from baseline to the study period for the BIO group; however, inpatient stays increased by 0.015 (*p* = 0.0014) and 0.004 (*p* = 0.0007) stays per patient for the CTS and IMS groups. ER visits increased slightly by 0.002 (*p* = 0.0024) for CTS and decreased by 0.004 (*p* < 0.0001) for IMS.

**Table 4:** Study period utilization and costs for patients with non-infectious uveitis by treatment group

	**CTS patients**^**a**^ **(*N*** **= 4,568)**	**IMS patients**^**b**^ **(*N*** **= 5,466)**	**BIO patients** **(*N*** **= 1,694)**
Total monthly healthcare resource utilization per patient, mean (SD)			
Inpatient stay (IP)			
Number of stays/patient	0.044 (0.34)	0.048 (0.30)	0.024 (0.19)^a,b^
Number of days/ hospitalized patient	3.678 (3.89)	3.621 (4.18)	3.862 (3.68) ^b^
Emergency room (ER)			
Number of visits/patient	0.030 (0.13)	0.030 (0.13)	0.030 (0.13)^a,b^
Office/outpatient (OP)			
Number of visits/patient	2.025 (1.81)	2.365 (1.95)	1.939 (1.55)
Pharmacy services			
Number of scripts/patient	3.015 (2.36)	4.106 (2.93)^a^	3.389 (2.66)^a,b^
Total monthly healthcare costs per patient, mean [median] (SD) (US$)	1,144 [382] (3,601)	1,759 [484] (5,474)^a^	2,689 [2,194] (2,912)^a,b^
Medical services (excluding study drugs)	902 [195] (3,445)	1,324 [196] (5,194)	716 [273] (2,005)^a,b^
Inpatient stay (IP)	307 [0] (2,497)	436 [0] (3,891)	183 [0] (1,444)^a,b^
Emergency room (ER)	21 [0] (148)	25 [0] (277)^a^	21 [0] (373)^a,b^
Office/outpatient (OP)	574 [185] (1,720)	863 [186] (2,482)	512 [252] (1,110)^a,b^
Pharmacy services (excluding study drugs)	227 [82] (576)	268 [85] (538)	201 [87] (398)
Total study drug costs	16 [7] (26)	167 [64] (380)^a^	1,772 [1,491] (1,532)^a,b^
Total costs excluding study drug costs	1,129 [365] (3,599)	1,592 [379] (5,383)	918 [466] (2,105)^a,b^

CTS, corticosteroids; IMS, immunosuppressive therapy; BIO, biologics. Chi-square tests were used to test differences in categorical variables. Wilcoxon tests were used to test differences in continuous variables. Healthcare costs and visits were calculated per-member-per-month (PMPM). Sample means and standard deviations were weighted by treatment months.^ a,b^Data with superscripted letter(s) indicates a significant difference (*P* < 0.05) from the uveitis subtype represented by that letter.

In the study period, BIO patients were associated with the highest monthly total healthcare costs. While all-cause monthly healthcare costs during the study period increased considerably from baseline in the BIO group (+US$1,250, *p* < 0.0001) compared to the CTS (+US$209, *p* < 0.0001) and IMS (+US$21, NS, *p* = 0.1075) groups, relatively sizable reductions in inpatient costs versus baseline occurred in the IMS (−US$256, *p* < 0.0001) and BIO groups (−US$225, *p* = 0.0033). All-cause monthly costs in the study period excluding study drugs were lowest in the BIO group. Differencing these costs versus the baseline resulted in cost increases in CTS (+US$194, *p* < 0.0001) and decreases in IMS (−US$146, *p* < 0.0001) and BIO (−US$521, *p* < 0.0001).

In sensitivity analysis requiring 90 (*N* = 2,341) or 30 (*N* = 19,426) days or more of continuous corticosteroid use to be in the CTS sample, all-cause monthly costs excluding study drugs increased + US$97 (*p* = 0.0002) and + US$257 (*p* < 0.0001) in CTS.

When excluding patients with rheumatoid arthritis, Crohn's disease, psoriasis, or ankylosing spondylitis, baseline and study costs were generally consistent with the overall analysis. Average costs excluding study drugs in the CTS (*N* = 4,169) sample increased (+US$226, *p* < 0.0001); in the IMS (*N* = 4,287) sample, they increased slightly (+US$56, *p* < 0.0001), and in BIO (*N* = 524), they decreased (−US$841, NS, *p* = 0.3519).

When using ICD-9-CM diagnosis codes more specific to posterior disease, average cost differences excluding study drugs between study and baseline periods increased (+US$176, *p* < 0.0001) in the CTS (*N* = 1,979) sample; increased (+US$133, *p* < 0.0001) in the IMS (*N* = 2,465) sample, and decreased (−US$768, *p* = 0.0152) in the BIO (*N* = 672) sample.

Lastly, when excluding patients who were not diagnosed by an ophthalmologist in order to increase the accuracy of the non-infectious uveitis diagnosis, baseline and study costs were also generally consistent with the overall analysis. Average costs excluding study drugs increased (+US$228, *p* < 0.0001) in the CTS (*N* = 3,732) sample; decreased slightly (−US$178, *p* < 0.0001) in the IMS (*N* = 4,144) sample; and decreased (−US$567, *p* < 0.0001) in the BIO sample (*N* = 1,323).

## Discussion

Little is known about the actual practice patterns of physicians treating uveitis. Our approach, using a retrospective analysis of a large claims data set, has advantages over previous research, which has generally relied on smaller samples that are often focused on the elderly, tertiary care centers, or a limited community population. Previous research has found that the types of cases seen in tertiary care centers or over limited geographies can vary based on disease etiology and anatomic location, limiting their generalizability [20]. Case series reports from subspecialty practices may not reflect what general ophthalmologists see and treat. The current study utilizes health insurance claims data from about one-quarter of the US population who are geographically diversely distributed and are cared for by hospital and community doctors alike, including inflammatory disorder ophthalmologists, general ophthalmologists, and other types of providers.

Findings indicate that the financial burden of chronic non-infectious uveitis is comparable to other medically and economically significant disease. Costs of treated patients with uveitis in the baseline and study periods were high for all groups relative to the average privately insured patient, whose average monthly (annual) spending in the USA was US$323 (US$3,868) in 2009 dollars [21]. Comparative study period costs per month in 2009 dollars for CTS, IMS, and BIO patients were US$1,144, US$1,759, and US$2,689, respectively. After taking into account healthcare cost inflation, we estimated that the average cost for non-infectious uveitis patients ranges from 3.1 to 8.3 times the costs of the average privately insured patient in 2009. These observed mean monthly healthcare costs for non-infectious uveitis are similar or higher than those for diabetes or hypertension patients (US$1,016 or US$723 in 2009 dollars) [22] in a privately insured population and similar or lower than those for cancer patients (US$2,649 for prostate cancer to US$9,225 for pancreatic cancer in 2009 dollars) in a private and Medicare population [23].

In this study, a substantial proportion of chronic uveitis patients received CTS alone, and a substantial majority of these patients were never observed to switch to IMS or BIO. While the CTS may have resolved the uveitis for some patients, average costs, utilization and rates of ophthalmological comorbidities increased during the CTS treatment episodes, indicating evidence of increased disease burden. Similar findings were evident in the sensitivity analyses requiring 90 or 30% days of continuous corticosteroids to be included in the CTS sample; in the latter sensitivity, *N* = 19,426 patients received CTS, 88% of whom had no evidence of receiving immunosuppressants or biologics later despite increased evidence of increased disease burden.

Nguyen et al. [10] found that the majority of physicians that treat a high volume of uveitis cases (primarily ophthalmologists) preferred high CTS doses to treat non-infectious uveitis and did not adhere to currently recommended guidelines for management of uveitis. Guidelines recommend the addition of IMS as a steroid-sparing agent if inflammation cannot be controlled with ≤10 mg/day of prednisone (or equivalent) within 3 months [8,24]. Additionally, our data showed no change in the treatment distribution of CTS versus IMS or BIO over 2003 to 2009, indicating that adoption of the guidelines was slow at best over this time period. Importantly, CTS therapy was associated with increases in hospital admissions and ER visits and all-cause costs. Continued use of CTS could be due to uncertainty regarding treating severe inflammation among community ophthalmologists, fear about side effects or skepticism about efficacy of IMS or BIO, inexpensive drug costs of CTS, or lack of awareness of the current guidelines.

This research also provides evidence that IMS and BIO therapy may be more effective at reducing ophthalmologic comorbidities related to uveitis than CTS. Further, treatment with BIO was associated with the largest reductions in non-study drug (i.e., non-biologic) costs and declines in hospital admissions and ER visits. Most IMS (82%) patients were never observed to switch to BIO in the data. Resistance to using biologics may be due to the lack of clinical trial evidence, the expense of the agents, perceptions about safety, and a lack of experience with using biologics.

Though not a focus of the study, we calculated simple measures of prevalence by counting the patients with uveitis based on diagnosis codes in 2003 to 2009, only in 2003, and only in 2008. Our simple prevalence estimate, in terms of patients who ever had a diagnosis of uveitis using the data, was about 201/100,000; using only those patients with diagnoses in 2003 and 2008, prevalence figures were 88 and 113/100,000. Gritz and Wong [13] estimated that their period prevalence, calculated as the mid-period prevalence in their one year study in Northern California, was 115.3 cases/100,000 persons. Reeves et al. [14] estimated that cumulative prevalence in a Medicare population of posterior uveitis ranged from 108 to 286/100,000. Our estimates are generally lower but in the same range as Reeves et al., potentially because we excluded patients over age 65 due to dual insurance issues with private supplemental insurance or Medicare.

Limitations arise from the nature of the administrative claims data used in this study. Claims data are designed for billing purposes rather than research, and measurement error via miscoding of conditions may occur [25,26]. Further, lack of clinical detail in both disease etiology and severity may lump heterogeneous patient groups into the same disease category. Further research may consider risk adjusting patients at baseline, which was not performed in this analysis. Patients in our study treated with CTS are likely to vary significantly in the severity of their uveitis, and BIO patients had the highest average Charlson comorbidity index (*p* = 0.0001 versus both). Patients receiving both corticosteroids and immunosuppressants are included in this analysis but their results are not separately broken out from the IMS group. Future research could examine the subgroups within our three treated patient samples. Further, we do not claim that the costs, inpatient or otherwise, in the paper are uveitis-caused costs. Rather, we describe costs of patients with uveitis who are on three different treatments. Accordingly, these costs could be due to unrelated reasons, potentially related to uveitis, or directly related to uveitis. Identifying the causality for cost types is a highly challenging research problem and not something we set out to do. Nevertheless, these data are appropriate for studying all-cause costs and utilization, as they contain diagnosis, testing, and procedure information, and represent payments paid by insurers to providers, and not charges, for these patients. Finally, claims data are available for patients with private insurance entering the healthcare system for treatment and thus capture only those individuals who have used the health system and had a claim for services.

## Conclusions

This analysis suggests that the burden of uveitis in the US privately insured population is economically significant. Patients treated with IMS or BIO were associated with improvements in rates of ophthalmologic comorbidities as well as reductions in non-study drug costs. Patients treated with IMS or BIO were also associated with improvements in rates of hospital admission and ER visits relative to CTS, which was associated with increases in these measures. Given the increase in comorbidity burden associated with CTS patients in the baseline and study comparison, there may be evidence that these patients are not appropriately treated.

## Abbreviations

BIO: Biologics; CTS: Corticosteroids; ER: Emergency room; IMS: Immunosuppressants; PMPM: Per-member-per-month.

## Competing interests

Chu has received honorarium from Analysis Group, Inc. which has received research funds from Novartis Pharmaceuticals Corporation. Johnson, Davis, Sorg, and Duh are employees of Analysis Group, Inc. Mallya is an employee of Novartis Pharmaceuticals Corporation.

## Authors’ contributions

DC, SJ, UM, MD, RS, and MSD designed the study. SJ, MD, RS, and MSD managed and analyzed the data. DC, SJ, MD, RS, and MSD interpreted the data and prepared the manuscript. All authors reviewed and approved the final manuscript.

## Supplementary Material

Additional file 1 Technical appendices. Click here for file

## References

[B1] Chang JH Wakefield D Uveitis: a global perspective Ocul Immunol Inflamm 2002 3 263 279 10.1076/ocii.10.4.263.15592 12854035

[B2] Suttorp-Schulten MS Rothova A The possible impact of uveitis in blindness: a literature survey Br J Ophthalmol 1996 3 844 848 doi:10.1136/bjo.80.9.844 10.1136/bjo.80.9.844 8962842 PMC505625

[B3] Darrell RW Wagener HP Kurland LT Epidemiology of uveitis: incidence and prevalence in a small urban community Arch Ophthalmol 1962 3 502 514 doi:10.1001/archopht.1962.00960030506014 10.1001/archopht.1962.00960030506014 13883604

[B4] Nussenblatt RB The natural history of uveitis Int Ophthalmol 1990 3 5–6 303 308 doi:10.1007/BF00163549 2249907 10.1007/BF00163549

[B5] Durrani OM Tehrani NN Marr JE Moradi P Stavrou P Murray PI Degree, duration, and causes of visual loss in uveitis Br J Ophthalmol 2004 3 1159 1162 doi:10.1136/bjo.2003.037226 10.1136/bjo.2003.037226 15317708 PMC1772296

[B6] Durrani OM Meads CA Murray PI Uveitis: a potentially blinding disease Ophthalmologica 2004 3 223 236 doi:10.1159/000078612 10.1159/000078612 15258410

[B7] Nguyen QD Callanazn D Dugel P Godfrey DG Goldstein DA Wilensky JT Treating chronic noninfectious posterior segment uveitis: the impact of cumulative damage. Proceedings of an expert panel roundtable discussion Retina 2006 3 suppl 1 16 17050954 10.1097/01.iae.0000250601.15893.5f

[B8] Jabs DA Rosenbaum JT Foster CS Holland GN Jaffe GJ Louie JS Nussenblatt RB Stiehm ER Tessler H Van Gelder RN Whitcup SM Yocum D Guidelines for the use of immunosuppressive drugs in patients with ocular inflammatory disorders: recommendations of an expert panel Am J Ophthalmol 2000 3 4 492 513 10.1016/S0002-9394(00)00659-0 11024423

[B9] Stanbury RM Graham EM Systemic corticosteroid therapy–side effects and their management Br J Ophthalmol 1998 3 6 704 708 10.1136/bjo.82.6.704 9797677 PMC1722622

[B10] Nguyen QD Hatef E Kayen B Macahilig CP Ibrahim M Wang J Shaikh O Bodaghi B A cross-sectional study of the current treatment patterns in noninfectious uveitis among specialists in the United States Ophthalmology 2011 3 1 184 190 doi:10.1016/j.ophtha.2010.03.029 10.1016/j.ophtha.2010.03.029 20678806

[B11] Imrie FR Dick AD Nonsteroidal drugs for the treatment of noninfectious posterior and intermediate uveitis Curr Opin Ophthalmol 2007 3 212 219 10.1097/ICU.0b013e3281107fef 17435428

[B12] Imrie FR Dick AD Biologics in the treatment of uveitis Curr Opin Ophthalmol 2007 3 481 486 10.1097/ICU.0b013e3282f03d42 18163000

[B13] Gritz DC Wong IG Incidence and prevalence of uveitis in Northern California. The Northern California Epidemiology of Uveitis Study Ophthalmology 2004 3 491 500 10.1016/j.ophtha.2003.06.014 15019324

[B14] Reeves SW Sloan FA Lee PP Jaffe GJ Uveitis in the elderly: epidemiological data from the National Long-term Care Survey Medicare Cohort Ophthalmology 2006 3 302 307 10.1016/j.ophtha.2005.10.008 16406541

[B15] Schneeweiss S Avorn JA Review of uses of health care utilization databases for epidemiologic research on therapeutics J Clin Epi 2005 3 323 337 10.1016/j.jclinepi.2004.10.012 15862718

[B16] Rein DB Zhang P Wirth KE Lee PP Hoerger TJ McCall N Klein R Tielsch JM Vijan S Saaddine J The economic burden of major adult visual disorders in the United States Arch Ophthalmol 2006 3 1754 1760 10.1001/archopht.124.12.1754 17159036

[B17] Hodge WG Incidence and prevalence of uveitis in Northern California: discussion by Ophthalmology 2004 3 500 doi:10.1016/j.ophtha.2003.10.017 10.1016/j.ophtha.2003.10.017 15019324

[B18] Deyo RA Cherkin DC Ciol MA Adapting a clinical comorbidity index for use with ICD-9-CM administrative databases J Clin Epidemiol 1992 3 6 613 619 10.1016/0895-4356(92)90133-8 1607900

[B19] Peterson AM Nau DP Cramer JA Benner J Gwadry-Sridhar F Nichol M A checklist for medication compliance and persistence studies using retrospective databases Value Health 2007 3 1 3 12 10.1111/j.1524-4733.2006.00139.x 17261111

[B20] McCannel CA Holland GN Helm CJ Cornell PJ Winston JV Rimmer TG Causes of uveitis in the general practice of ophthalmology: UCLA Community-Based Uveitis Study Group Am J Ophthalmol 1996 3 35 46 8554079 10.1016/s0002-9394(14)70532-x

[B21] Bundorf MK Royalty A Baker LC Health care cost growth among the privately insured Health Aff (Millwood) 2009 3 5 1294 1304 Costs inflation adjusted to 2009 dollars using the medical Consumer Price Index. doi:10.1377/hlthaff.28.5.1294 10.1377/hlthaff.28.5.1294 19738244

[B22] Laliberté F Bookhart BK Vekeman F Corral M Duh MS Bailey RA Piech CT Lefebvre P Direct all-cause health care costs associated with chronic kidney disease in patients with diabetes and hypertension: a managed care perspective J Manag Care Pharm 2009 3 4 312 322 Costs inflation adjusted to 2009 dollars using the medical Consumer Price Index 19422271 10.18553/jmcp.2009.15.4.312 PMC10437749

[B23] Chang S Long SR Kutikova L Bowman L Finley D Crown WH Bennett CL Estimating the cost of cancer: results on the basis of claims data analyses for cancer patients diagnosed with seven types of cancer during 1999 to 2000 J Clin Oncol 2004 3 17 3524 3530 doi:10.1200/JCO.2004.10.170, Costs inflation adjusted to 2009 dollars using the medical Consumer Price Index 10.1200/JCO.2004.10.170 15337801

[B24] Standardization of Uveitis Nomenclature (SUN) Working Group Standardization of uveitis nomenclature for reporting clinical data: results of the first international workshop Am J Ophthalmol 2005 3 509 516 doi:10.1016/j.ajo.2005.03.057 16196117 10.1016/j.ajo.2005.03.057 PMC8935739

[B25] Epstein AM Cumella EJ Capitation payment: using predictors for medical utilization to adjust rates Health Care Financ Rev 1988 3 51 69 10312821 PMC4192907

[B26] Whittle J Steinberg EP Anderson GF Herbert R Accuracy of Medicare claims data for estimation of cancer incidence and resection rates among elderly Americans Med Care 1991 3 1226 1236 10.1097/00005650-199112000-00005 1745080

