# Supplementary material for: Healthcare costs and utilization for privately insured patients treated for non-infectious uveitis in the USA
Source: J Ophthalmic Inflamm Infect. 2013 Nov 6;3:64. doi: 10.1186/1869-5760-3-64 (PMC3830557; doi:10.1186/1869-5760-3-64)
Supplement: Additional file 1 — Technical appendices. [file 1869-5760-3-64-S1.docx]

**Additional file 1 Table 1. ICD-9-CM Diagnosis Codes Used to Identify Non-infectious Uveitis**

| **Description** | **ICD-9-CM Code** |
| --- | --- |
| Acute endophthalmitis | 360.01* |
| Panophthalmitis | 360.02* |
| Chronic endophthalmitis | 360.03* |
| Other endophthalmitis | 360.1* |
| Sympathetic uveitis | 360.11* |
| Panuveitis | 360.12* |
| Exudative retinitis | 362.12* |
| Retinal (peri) vasculitis | 362.18* |
| Focal choroiditis and chorioretinitis, unspecified | 363.00* |
| Focal choroiditis and chorioretinitis, juxtapapillary | 363.01* |
| Focal choroiditis and chorioretinitis, posterior pole | 363.03* |
| Focal choroiditis and chorioretinitis, peripheral | 363.04* |
| Focal retinitis and retinochoroiditis, juxtapapillary | 363.05* |
| Focal retinitis and retinochoroiditis, macular or paramacular | 363.06* |
| Disseminated chorioretinitis, unspecified | 363.10* |
| Disseminated choroiditis and chorioretinitis, posterior pole | 363.11* |
| Disseminated choroiditis and chorioretinitis, peripheral | 363.12* |
| Disseminated choroiditis and chorioretinitis, generalized | 363.13* |
| Disseminated retinitis and retinochoroiditis, metastatic | 363.14* |
| Disseminated retinitis and retinochoroiditis, pigment | 363.15* |
| Chorioretinitis, unspecified | 363.20* |
| Pars planitis/posterior cyclitis | 363.21 |
| Harada’s disease (uveomeningeal syndrome) | 363.22* |
| Acute and subacute iridocyclitis | 364.0x |
| Chronic iridocyclitis | 364.1x |
| Certain types of iridocyclitis | 364.2 |
| Fuch’s heterochromic cyclitis | 364.21 |
| Glaucomatocyclitis crisis | 364.22 |
| Lens-induced iridocyclitis | 364.23 |
| Vogt–Koyanagi syndrome | 364.24* |
| Unspecified iridocyclitis uveitis NOS | 364.3 |

* Indicates code used in posterior specific code sensitivity analysis.

**Additional file 1 Table 2: List of Corticosteroids, Immunosuppressants, or Biologics Included in Analysis**

| **Class** | **Drug** |
| --- | --- |
| Corticosteroids | prednisone |
|  | prednisolone |
|  | dexamethasone |
|  | triamcinolone |
|  | dexamethasone |
|  | hydrocortisone |
|  | methylprednisolone |
|  | betamethasone |
|  | prednisolone |
|  | dexamethasone |
|  | fluorometholone |
|  | loteprednol |
|  | difluprednate |
|  | medroxyprogesterone |
|  | medrysone |
|  | rimexolone |
|  | fluocinolone |
|  | dexamethasone |
| Immunosuppressants | azathioprine |
|  | methotrexate |
|  | mycophenolate |
|  | mycophenolic acid |
|  | tacrolimus |
|  | chlorambucil |
|  | cyclophosphamide |
|  | cyclosporine |
|  | tacrolimus |
| Biologics | etanercept |
|  | adalimumab |
|  | infliximab |
|  | ustekinumab |
|  | interferon alpha-2a |
|  | interferon beta-1a |
|  | daclizumab |
|  | rituximab |

Additional file 1 Table 3: Ophthalmologic Comorbidities Related to Uveitis

| **Comorbidity** | **ICD-9-CM Code** |
| --- | --- |
| **Retinal detachments** | 361.xx |
| Retinal detachment with retinal defect | 361.0 |
| Retinoschisis and retinal cysts | 361.1 |
| Serous retinal detach | 361.2 |
| Retinal defects without detachment | 361.3 |
| Other forms of retinal detachment | 361.8 |
| Unspecified retinal detach | 361.9 |
| **Glaucoma** | 365.xx |
| Borderline glaucoma (glaucoma suspect) | 365.0 |
| Open-angle glaucoma | 365.1 |
| Primary angle-closure glaucoma | 365.2 |
| Corticosteroid-induced glaucoma | 365.3 |
| Glaucoma associated with congenital anomalies dystrophies and systemic syndromes | 365.4 |
| Glaucoma associated with disorders of the lens | 365.5 |
| Glaucoma associated with other ocular disorders | 365.6 |
| Other specified forms of glaucoma | 365.8 |
| Other specified forms of glaucoma | 365.9 |
| **Cataract** | 366.xx |
| Infantile juvenile and presenile cataract | 366.0 |
| Senile cataract | 366.1 |
| Traumatic cataract | 366.2 |
| Cataract secondary to ocular disorders | 366.3 |
| Cataract associated with other disorders | 366.4 |
| After-cataract | 366.5 |
| Other cataract | 366.8 |
| Unspecified cataract | 366.9 |
| **Visual disturbances** | 368.xx |
| Subjective visual disturbances | 368.1 |
| Other disorders of binocular vision | 368.3 |
| Visual field defects | 368.4 |
| Color vision deficiencies | 368.5 |
| Night blindness | 368.6 |
| Other specified visual disturbances | 368.8 |
| Unspecified visual disturbance | 368.9 |
| **Blindness** | 369.xx |
| Profound vision impairment, both eyes | 369.0 |
| Moderate or severe vision impairment better eye;  profound vision impairment of lesser eye | 369.1 |
| Moderate or severe vision impairment, both eyes | 369.2 |
| Unqualified visual loss both eyes | 369.3 |
| Legal blindness as defined in USA | 369.4 |
| Profound vision impairment one eye | 369.6 |
| Moderate or severe vision impairment one eye | 369.7 |
| Unqualified visual loss one eye | 369.8 |
| Unspecified visual loss | 369.9 |
| **Cystoid macular degeneration** | 362.53 |
| **Other complications** |  |
| Phthisis bulbi | 360.4 |
| Chorioretinal scars | 363.3 |
| Optic atrophy | 377.1 |
| Optic neuritis | 377.3 |
| Other disorders of optic nerve | 377.4 |
| Hypotony | 360.3 |
| Band-shaped keratopathy | 371.4 |
